# Human Dendritic Cells Activated via Dectin-1 Are Efficient at Priming Th17, Cytotoxic CD8 T and B Cell Responses

**DOI:** 10.1371/journal.pone.0013418

**Published:** 2010-10-18

**Authors:** Sudhanshu Agrawal, Sudhir Gupta, Anshu Agrawal

**Affiliations:** Division of Basic and Clinical Immunology, Department of Medicine, University of California Irvine, Irvine, California, United States of America; Beijing Institute of Infectious Diseases, China

## Abstract

**Background:**

Dendritic cells capture antigens through PRRs and modulate adaptive immune responses. The type of adaptive immune T cell response generated is dependent upon the type of PRR activated by the microbes. Dectin-1 is a C-type lectin receptor present on dendritic cells.

**Methodology/Principal Findings:**

Here we show that selective dectin-1 agonist Curdlan can activate human DCs and induce the secretion of large amounts of IL-23, IL-1β, IL-6 and low levels of IL-12p70 as determined by ELISA. The Curdlan-stimulated DCs are efficient at priming naïve CD4 cells to differentiate into Th17 and Th1 cells. Furthermore, these CD4 T cells induce differentiation of B cells to secrete IgG and IgA. In addition, Curdlan-stimulated DCs promote the expansion and differentiation of Granzyme and perforin expressing cytotoxic T lymphocyte that display high cytolytic activity against target tumor cells in vitro.

**Conclusions/Significance:**

These data demonstrate that DCs stimulated through Dectin-1 can generate efficient Th, CTL and B cell responses and can therefore be used as effective mucosal and systemic adjuvants in humans.

## Introduction

Cells of the innate immune system such as dendritic cells (DCs) detect and respond to pathogens through the expression of pattern recognition receptors (PRRs). PRRs can recognize conserved molecular components or patterns of the pathogens. Examples of PRRs include Toll-like receptors (TLRs), RIG-I like receptors, and Nod-like receptors [1], [2]. Besides these, a new class of PRRs, the C-type lectin receptor family has also emerged as a major sensor of pathogens. C-type lectins recognize carbohydrate moieties on bacteria and fungi [3]–[6]. Exposure of DCs to ligands of all these PRRs results in production of cytokines that modulate the type of T cell response and functions [1], [7]–[8]. Upon interaction with DCs, CD4^+^ T cells can differentiate into a variety of effector and regulatory subsets, including classical Th1 cells and Th2 cells, follicular helper T cells, induced regulatory T cells and the more recently defined Th17 cells [7], [8]. The nature of the cytokines produced by DCs in response to various ligands dictates the type of Th cell responses. For example, IL-12p70 secretion by DCs polarizes towards Th1 cells [9] while the production of IL-23 along with IL-1β from DCs leads to the generation of Th17 cells [10], [11]. Our previous studies have also shown that engagement of different TLRs on DCs produces divergent type of adaptive immune responses. Ligation of TLR4 and TLR5 on DCs by LPS and Flagellin resulted in the production of IL-12p70, biasing the Th response towards Th1. Engagement of TLR2 on DCs via Pam3cys on the other hand generates a Th2 type of response. However, simultaneous engagement of TLR 2/6 and Dectin-1 by Zymosan polarized the Th cell response towards Th0 or Treg [12]–[14]. DCs are thus capable of modulating the nature of T cell responses through their cytokine secretion which in turn is dependent on the type of receptor that is activated.

Phagocytes, such as macrophages and DCs, express several types of C-type lectin receptors on their cell surfaces for antigen capture. Dectin-1 is an example of C-type lectin receptor that recognizes fungal β-glucan and is critical for its biological effects. β-glucans are carbohydrate polymers found primarily in the cell walls of fungi, but also in plants and some bacteria. The Dectin-1 agonist, β-glucan acts as an adjuvant as well as an immunotherapeutic agent in the treatment of a number of diseases [3]–[6]. The immune mechanisms responsible for the success of β-glucans in immunotherapy are still unclear. Recent studies in mice suggest that β-glucans bind to dectin-1 on phagocytes and signal via Syk kinase independent of the TLR pathway. They prime primarily Th17 responses [15]. Recently it was observed that DCs activated via Dectin can convert Treg to IL17 producing cells [16] Furthermore, they also prime cytotoxic T-lymphocyte (CTL) responses and mount potent CTL responses [17]. Dectin-1 also induces antibody production in rodents [18]. It is not known if a similar mechanism exists in humans. In the present study we sought to determine mechanism of action β-glucans in humans by determining the response in human DCs. It is essential to understand fully the nature of adaptive immune responses induced in humans by these stimuli in order to harness their powerful modulating properties to tailor immune responses against a specific pathogen or disease.

## Results

### Dectin-1 agonist, Curdlan activates dendritic cells to induce a distinct profile of cytokine secretion as compared to Zymosan and LPS

**Figure 1:**
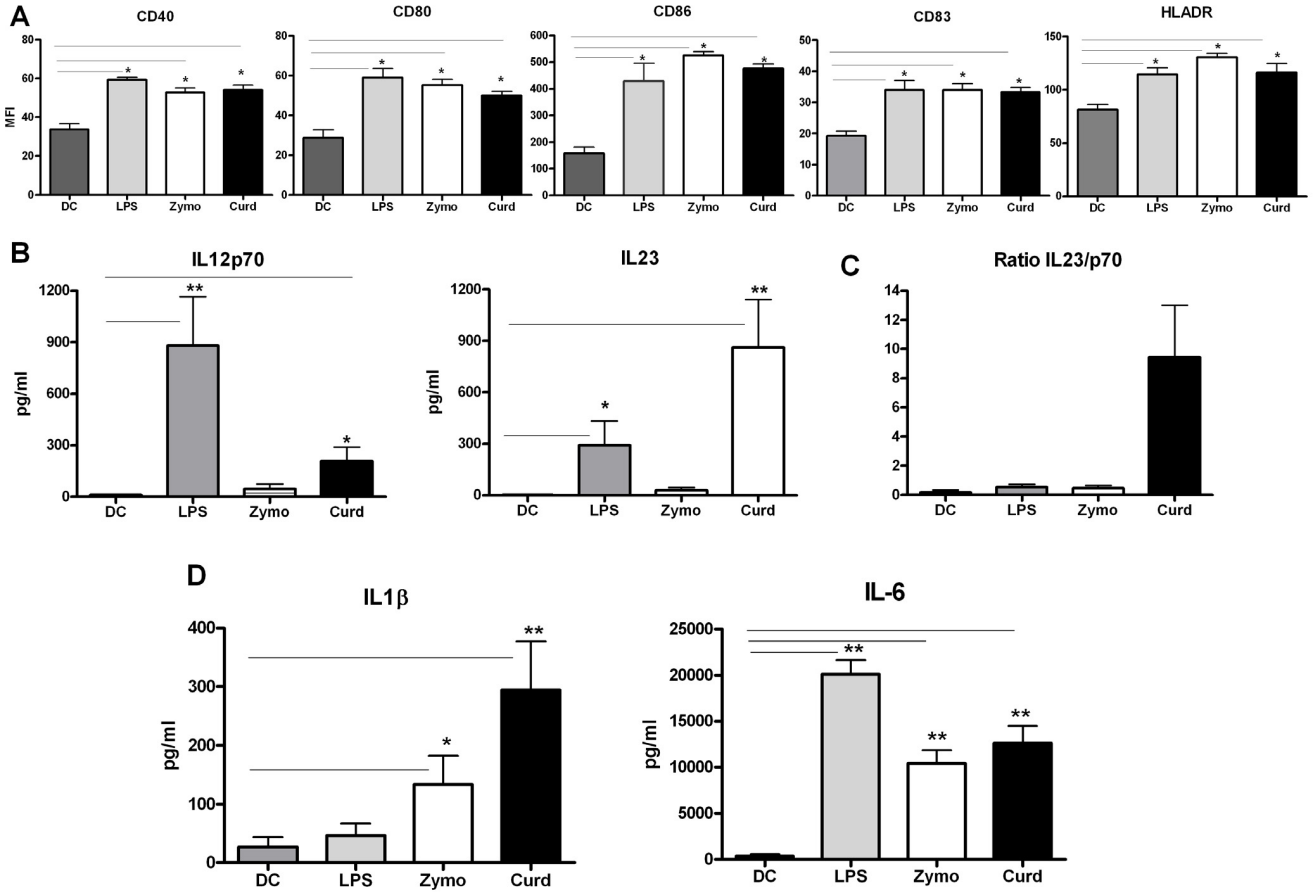
Dectin-1 agonist, Curdlan activates dendritic cells to induce a distinct profile of cytokine secretion. DCs were activated with Curdlan, Zymosan and LPS for 48 h and upregulation of surface markers and secretion of cytokines was determined. **A.** Bar graph depicts the mean fluorescence intensity [MFI] of CD40, CD80, CD86, CD83 and HLADR on DCs after activation. Figure is mean ± S.E. of 5 such experiments. **B.** Bar graphs depict the pg/ml level of IL-23 and IL-12p70 secreted by DCs in response the stimuli. **C.** Bar graph depicts the ratio of IL-23/IL-12p70 of the same. **D.** Bar graphs depict the pg/ml level of IL-1β and IL-6 of the same. Figure is mean ± S.E. of 10 such experiments. * Significant (<0.05), ** highly significant (<0.005).

Our previous studies had shown that stimulation of DCs by Yeast cell wall Zymosan, a TLR 2/6 and Dectin-1 stimulus polarized the CD4 T cell response towards T- regulatory type [14] while E.coli LPS, a TLR4 agonist polarized it towards Th1 type response [12]. Here we wanted to delineate further the difference between TLR 2/6 responses from that of Dectin-1 since recent studies [4], [15] had indicated that the two responses are different. Curdlan was chosen as a Dectin-1 agonist as Zymosan signals through both TLR2 and Dectin-1. Curdlan is a linear β-1, 3-glucan of ∼500-mers derived from the bacterium *Alcaligenes faecal. var. myxogenes* We examined the activation of DCs in response to these stimuli. Initial experiments were performed to determine the optimal concentrations for Zymosan and Curdlan that induced the secretion of IL-23 or IL-12p70. Zymosan at 40 µg/ml and Curdlan at 20 µg/ml were found to be the optimal dose for the present study (Figure S1). The concentration of LPS [1 µg/ml] used in these studies was chosen based on our previous studies [12]–[14]. To study the effect of different agonists on DCs, immature DCs were cultured in the presence of, Zymosan, Curdlan and E.coli LPS. All stimuli induced maturation of DCs within 48 h as evidenced by up-regulation of costimulatory and maturation markers CD40, CD80, CD86, CD83 and HLADR (Figure 1A). There was no significant difference (p>0.05) in the level of activation induced by all three stimuli.

Next, we investigated the cytokines secreted by these stimulated DCs. The cytokine response was remarkably different between the various stimuli. As shown in Figure 1B, a distinct profile of IL-12 family cytokines was observed. Stimulation with Curdlan favored IL-23 production (IL-23∼900 pg/ml vs. IL-12p70∼200 pg/ml) whereas stimulation with LPS resulted in high IL-12p70 production (IL-12p70∼800 pg/ml vs. IL-23∼300 pg/ml). Zymosan was a weak inducer of both IL-23 (∼40 pg/ml) and IL-12p70 (∼40 pg/ml). That Curdlan-stimulated DCs biased the DC cytokine secretion towards IL-23 was also evident when we determined the ratio of IL-23/IL-12p70. The ratio was more than 4 for Curdlan but less than 1 for both LPS and Zymosan (Figure 1C). Besides IL-12 and IL-23, these stimuli also induced the production of IL-1β and IL-6 from DCs. While Curdlan and Zymosan induced IL-6 production was not significantly different (Figure 1D), LPS induced significantly higher level (p<0.05) of IL-6 (Figure 1D). The secretion of IL-1β was significantly different between all three stimuli with Curdlan inducing the maximum level (∼300 pg/ml) followed by Zymosan (∼150 pg/ml) and LPS (∼50 pg/ml). Taken together these data demonstrate that stimulation of DCs through Curdlan induces distinct profile of cytokine secretion with high IL-23, IL-1β and IL6, and relatively low levels of IL-12p70.

### Stimulation of Dectin-1 on DCs biases the Th cell response primarily towards Th17

**Figure 2:**
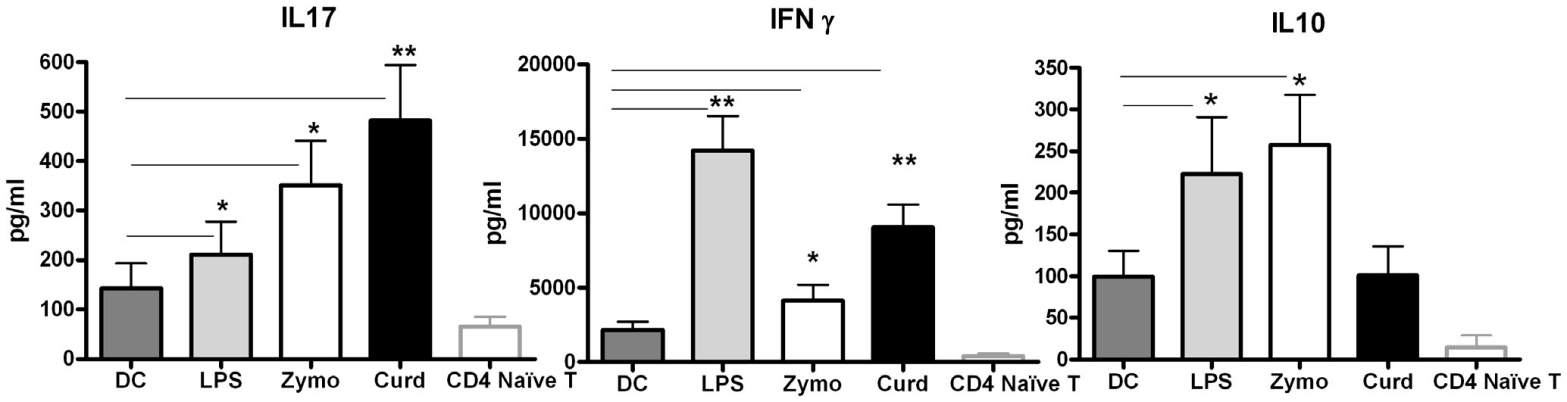
Stimulation of Dectin-1 on DCs biases the Th cell response primarily towards Th17. DCs activated with Curdlan, Zymosan and LPS were cocultured with naïve CD4 T cells for seven days. Cytokine secretion was determined by ELISA. Bar graphs depict the level of IL-17, IFN-γ and IL-10 secreted by CD4 T cells. Figure is mean ± S.E. of 10 such experiments. * Significant (<0.05), ** highly significant (<0.005).

A large body of evidence indicates that the nature of cytokine secretion by DCs dictates the polarization of Th cell responses towards Th1, Th2, Treg or Th17. High IL-23 and IL-1β favor IL-17 production from Th cells while high IL-12p70 favors IFN-γ production [7], [8]. Therefore, given the distinct profile of cytokine secretion by DCs in response to Curdlan, Zymosan and LPS, we explored its effect on Th cell responses. DCs were cultured with various stimuli as described in Figure 1. Two days after culture they were washed and cultured together with purified, naïve CD4 T cells (CD4+, CD45RA+) cells for seven days to allow differentiation of naïve T cells towards Th17 or Th1. We observed (Figure 2) that both Curdlan and Zymosan stimulated DCs biased the Th cell response towards Th17 and Th1 (Curdlan induced ∼500 pg/ml IL-17 and 8500 pg/ml IFN- γ), Zymosan induced ∼350 pg/ml IL-17 and ∼4000 pg/ml IFN-γ). Furthermore, Zymosan also induced significantly high levels (p<0.05) of IL-10 (∼260 pg/ml) as compared to Curdlan (∼50 pg/ml) (Figure 2). In contrast, LPS stimulated DCs induced a more Th1 type of response (∼200 pg/ml IL-17 and 14000 pg/ml IFN-γ, Figure 2). Recently IL-17 secretion by Curdlan stimulated DCs was also observed by Higashi et al [19] when they stimulated DCs with Curdlan concentration of 1 ng/ml much lower concentration than used by us. However, they were able to detect IL-17 only on restimulation of T cells with anti CD3 and anti-CD28 suggesting that the levels of IL-17 secreted were very low. Here we have used a concentration of Curdlan which led to comparable level of DC activation and cytokine secretion as Zymosan and LPS. In summary, these data suggest that high IL-23 and IL-1β secretion by Curdlan stimulated DCs induces both Th17 and Th1 cells. Compared to Curdlan, Zymosan stimulated DCs with low IL-23 and IL-1β induced a low Th17, even lower Th1 and high IL-10 type of T cell response. LPS stimulated DCs induced primarily a Th1 type of T cell response as before [12]–[14].

### CD4 T cells primed with Curdlan stimulated DCs induce IgG and IgA secretion in B cells

**Figure 3:**
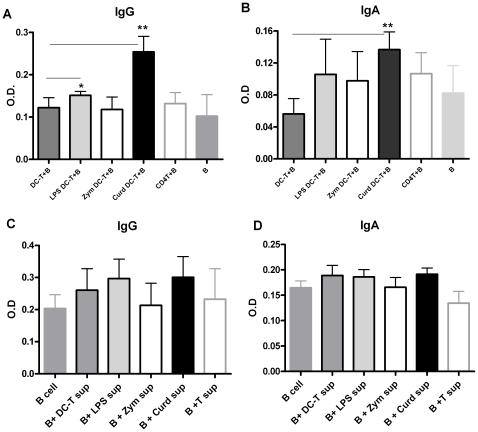
Dectin-1 stimulated DCs prime CD4 T cells to induce IgG and IgA in B cells. Naïve T cells primed by DCs as in figure 2 were cultured with allogeniec, purified B cells for two weeks. IgG and IgA secretion was determined by ELISA. **A.** Bar graph depicts the level of IgG in the supernatant. **B.** Bar graph depicts the level of IgA in the supernatant. Figure is mean ± S.E. of 4 such experiments. * Significant (<0.05), ** highly significant (<0.005).**C.** Bar graphs depict the level of IgG in the supernatant after culture of B cells with DC-T cell supernatant. **D.** Bar graphs depict the level of IgA in the supernatant after culture of B cells with DC-T cell supernatant.

Next, we determined the influence of the primed CD4 T cells with different Th cytokine responses, on the induction of various types of Immunoglobulin's from B cells. Naïve CD4 T cells were primed with DCs stimulated with LPS, Zymosan and Curdlan as above. Seven days later the T cells were collected and cultured with allogeneic B cells. Two weeks later, supernatants were collected and analyzed for the secretion of IgG and IgA. We observed that Curdlan exposed DC primed CD4 T cells induced significantly higher amount (p<0.05) of IgG and IgA secretion compared to all other groups (Figure 3). LPS exposed DC primed T cells also induced IgG and IgA albeit to a lower extent than Curdlan. Zymosan exposed DC primed T cells did not induce significant (p>0.05) IgG secretion over unstimulated DC primed T cells. However, they did induce IgA secretion compared to controls though the difference was not significant (p>0.05).

To investigate if the secretion of Ig by B cells required cell to cell contact with T cells or the cytokines secreted by T cells were sufficient for Ig induction, B cells were cultured in the presence of supernatants obtained from stimulated DC-T cell cultures (as in figure 2) for 14 days. Concentration of IgG and IgA in the culture was determined as described. Zymosan, Curdlan and LPS stimulated DC-T supernatants alone did not induce significant levels of IgG or IgA over unstimulated DC-T cell supernatant (Figure 3Cand D). This suggests that T and B cell contact is essential to drive the Ig production.

### Curdlan and Zymosan induce Granzyme expressing cytotoxic CD8 T cells

**Figure 4:**
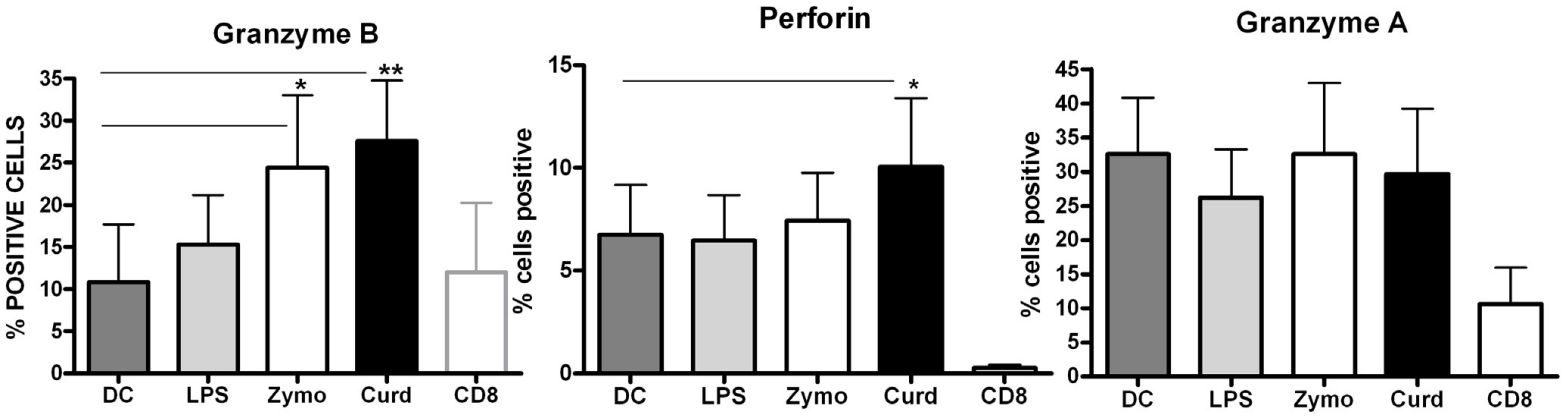
Curdlan and Zymosan induce Granzyme expressing cytotoxic CD8 T cells. DCs activated with Curdlan, Zymosan and LPS were cocultured with CD8 T cells for seven days. Granzyme A, granzyme B and perforin induction was determined by flow cytometry. Bar graphs depict the percentage of CD8 T cells expressing Granzyme B, perforin and Granzyme A. Figure is mean + S.E. of 5 such experiments. * Significant (<0.05), ** highly significant (<0.005).

In addition to influencing CD4 T cell responses, DCs also induce the generation of cyotoxic CD8 T cell responses. To explore this, we determined the effect of Dectin-1 stimulated DC on CD8 T cells. DCs cultured for 48 h with various stimuli were washed and cultured with purified CD8 T cells. Seven days later the cells were collected and stained for intracellular granzyme A, granzyme B and perforin. DCs stimulated with all three stimuli led to the induction of granzyme B positive CD8 T cells (Figure 4). However, Curdlan stimulated DCs induced the maximal number of granzyme B positive CD8 T cells (∼28%) followed closely by Zymosan (∼25%) while LPS stimulated DC induced ∼15%. In contrast to granzyme B, only Curdlan stimulated DCs induced significant levels (p<0.05) of perforin in CD8 T cells (Figure 4). Granzyme A was not induced at significant levels in any of the groups (Figure 4). These data suggest that stimulation of DCs through Dectin-1 is highly effective in priming cytotoxic T cell responses.

### Curdlan stimulated DCs prime CD8 T cells with higher cytolytic activity compared to Zymosan and LPS

**Figure 5:**
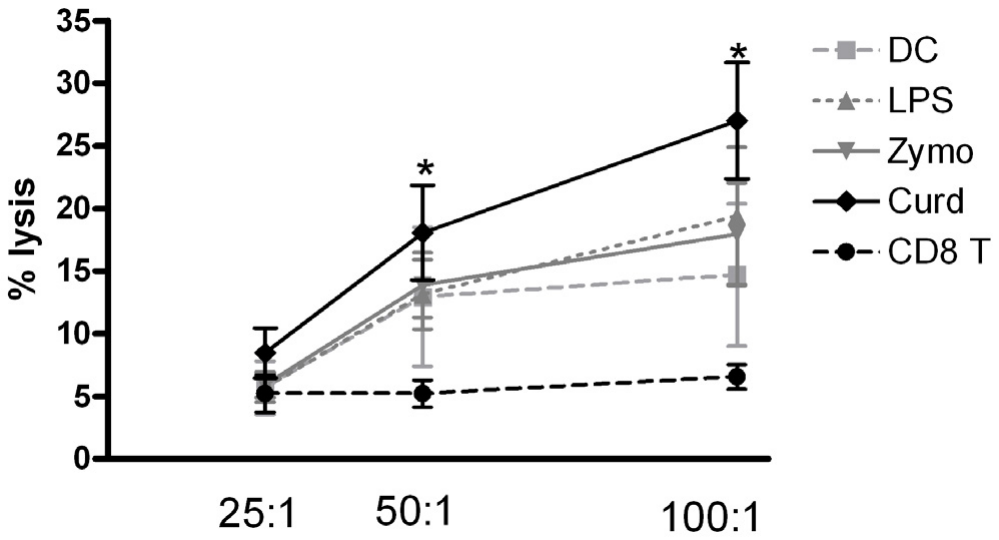
Curdlan stimulated DCs prime CD8 T cells with higher cytolytic activity compared to Zymosan and LPS. CD8 T cells generated as described in methods were incubated with PC3 target cells for 4 h. Percent lysis was determined by flow cytometry. Data represent mean ± S.E.M. of percent specific lysis of PC3s at indicated Effector:Target in 4 different experiments. * Significant (<0.05) as compared unstimulated DC -CD8 T cells.

We further determined the ability of these Granzyme expressing CD8 T cells to lyse tumor cells. PC3, the prostate cancer cell line (American Type Culture Collection, Manassas, VA) was used as the source of tumor cells. PC3 specific CD8 T cells were generated as described in methods. To determine the lysis, purified CD8 T cells (effectors) were co cultured with CFSE labeled PC3 cells (target) at target: effector ratios 25∶1, 50∶1, and 100∶1. Four hours later 7-AAD was added to the cells to stain for dead cells. Controls included CFSE-stained PC3 cells without effectors and 7AAD and CFSE-stained PC3 cells. Analysis was performed by gating on the target cells and measuring the 7AAD-negative vs. 7AAD-positive cells. Cells positive for both 7-AAD and CFSE were considered lysed. Percentage of cytotoxicity was calculated by the following equation: (7AAD-positive CFSE-positive cells/total number of CFSE-positive cells)×100. As seen in Figure 5, Curdlan stimulated DCs primed CD8 T cells with higher cytolytic activity (∼25% lysis at 100∶1 effector: target) ratio as compared to LPS and Zymosan stimulated DCs (∼17% lysis at 100∶1 effector: target). Similar results were observed at lower effector target ratios also. These data clearly suggest that stimulation of DCs through Dectin-1 alone induces highly cytolytic CD8 T cells while stimulation through a combination of TLR 2/6 and dectin-1 is not as effective.

## Discussion

DCs act as a bridge between the innate and the adaptive immune responses by sensing pathogens through PRRs and inducing adaptive immune responses [7], [8], [20], [21]. Though the induction and modulation of T cell responses via engagement of TLRs is well established [1], [3] nevertheless, there is limited information regarding the ability of non-TLR PRRs to induce and direct T cell responses. Dectin-1 is amongst the few non-TLR PRRs that can activate DCs and induce T cell responses [2], [16], [22]. Studies have shown that DCs activated selectively via the dectin-1 acquire effector functions similar to DCs stimulated by TLR agonists. They up-regulate costimulatory molecules, produce TNF-α, IL-6, IL-2, IL-10, and IL-23, and are able to prime CD4^+^ T cells and instruct their differentiation into IFN-γ and IL-17 producing T-helper cells [8], [23]. Adjuvants that stimulate the dectin-1 pathway also promote antibody responses in vivo. The dectin-1 PRR can thus couple pathogen sensing to adaptive immune T and B cell responses.

Most of these studies have been performed in mice [15]–[18], [24] and little is known about Dectin-1 responses in humans. It is essential to understand the Dectin-1 immune response in humans in order to exploit it for human use. Our data indicates that the responses of Dectin-1 in human DCs though essentially similar to that found in mouse also show subtle differences. Stimulation of DCs with Dectin-1 ligand, Curdlan led to the activation of DCs (Figure 1A) as in mice. We also observed that DCs activated with Curdlan were highly efficient producers of IL-23 (Figure 1B) as in murine studies however, in contrast to mice DCs, human DCs also induced the production of IL-12p70 though the levels induced were lower than that of IL-23 (Figure 1B and C). In addition to IL-12p70 and IL-23, stimulation of DCs with Curdlan also induced high levels of IL-1β (Figure 1D). This was primarily through Dectin-1 receptor since simultaneous engagement of TLR2 with Dectin-1 by Zymosan led to very low levels of production of all these cytokines. IL-1β is a central orchestrator of immunity against various classes of pathogens [25]–[31], and a key trigger of inflammatory diseases [32]. IL-23, especially in synergy with IL-1β, plays an essential role in the induction or expansion of murine and human Th17 cells. Indeed, culture of Curdlan stimulated DCs with naïve CD4 T cells sustained their differentiation towards Th17 cells as well as IFN-γ producing Th cells (Figure 2). These results are not surprising, as in humans, a substantial proportion of IL-17 producers are found to express IFN-γ [33]. Higashi et al [19] have also observed IL-17 secretion by Curdlan activated DCs though they did not observe IFN-γ secretion. This may be a due to differences in the concentration of Curdlan used to stimulate DCs. The IL-17 produced in response to dectin-1 stimulation is critical in providing protection against a range of fungal pathogens [27], [34]. Recently, Ferwarda et al [35] observed that in few patients more susceptible to mucocutaneous fungal infections there was an early stop codon mutation in Dectin-1 receptor which led to poor expression of the receptor resulting in poor β-glucan binding and subsequent impaired cytokine production.

There is very little information in the literature regarding the type of B cell immunoglobulin response induced by CD4 T cells expressing both IL-17 and IFN-γ. It has been suggested that IL-17 producing Th cells should result in the induction of IgA since IL-17 provides protection at mucosal surfaces. We observed that indeed Curdlan DC primed CD4T cells induced secretion of high levels of both IgA and IgG (Figure 3A and B) which is in keeping with T cell cytokine profile of high IL-17 and IFN-γ. Zymosan DC primed CD4 T cells induced only low amounts of IgA and no IgG. This may be because along with IL-17 and IFN-γ the Zymosan DC primed CD4 T cells also secreted high amounts of IL-10 (Figure 2). Activation of B cells in the presence of supernatants containing IL-17 did not lead to Ig secretion by B cells (Figure 3C and D) suggesting that cell to cell contact between Curdlan DC activated T cells and B cells is required for the IgG and IgA secretion. These observations suggest that in addition to IL-17, Curdlan DC stimulated T cells also provide contact dependent signals to induce IgA and IgG secretion from B cells.

Besides priming Th17 responses, Curdlan-stimulated human DCs were extremely efficient at priming cytotoxic CD8 T cells expressing Granzyme B and perforin as compared to both LPS and zymosan (Figure 4). This is similar to what was observed with murine DCs [15]–[17]. These perforin and granzyme B expressing cytotoxic T lymphocytes were highly cytolytic towards tumor cells (Figure 5) in vitro. The induction of Th17 and cytolytic CD8 T cells by Curdlan stimulated DCs may explain its effectiveness in cancer treatment. Beta-glucans or Curdlan are biological response modifiers already in use as alternative cancer treatment [36]–[40]. β-glucans are given subcutaneously for treating and reducing the size of skin tumors [41]. This is due to their ability to accelerate recovery in irradiated animal specimens, even when it is given after the radiation dose. A recent study [42] demonstrated that repeated immunizations with two MHC class-I restricted peptides derived from the tumor antigen survivin combined with oral co-administration of β-glucan could significantly diminish intradermal tumor growth, whereas peptide vaccination alone failed to control tumor growth. Further evidence come from the fact that DCs from human myeloma tumor bed are potent inducers of Th17 cells [33]. However, it is not known if these DCs can induce cytolytic CD8 T cell responses.

In summary, we have shown that C-type lectin receptors such as Dectin-1 are not just antigen uptake receptors but also modulators or initiators of adaptive immune responses. These Curdlan primed human DCs are efficient APCs for the induction of Th17 cells, cytolytic CD8 T cells and B cell antibody responses. The capacity of Curdlan-stimulated human DCs to induce differentiation of these cells makes them attractive target for manipulations in clinic. DCs are currently under intensive investigation as adjuvant for immunotherapy against cancer and other diseases. They are also being manipulated to expand T cells for adoptive transfer in diseases. Targeting the induction of Th17 cells and IgA antibodies may prove valuable in the induction of mucosal immunity and use of Curdlan and other β-glucans as mucosal adjuvants. This is particularly important, because though there exists a very long list of adjuvants for systemic immunization, the number of mucosal adjuvants is far more limited. Furthermore, Curdlan- stimulated DCs can also serve as efficient tools to prime tumor specific cytotoxic T cell responses. IL-17 secretion in response to Dectin-1 has also been implicated in driving autoimmunity [45]. Arthritis could be induced in pathogen free SKG mice after treatment with zymosan or purified β-glucans. The progression of arthritis could be inhibited by blocking Dectin-1 [46]. Furthermore, blockage of Dectin-1 could prevent experimental autoimmune uveoretinitis, a Th1/Th17 disease [4]. High expression of Dectin-1 mRNA was also observed in patients with psoriais [47]. These studies imply that dectin-1 plays a pivotal role in the innate immune system and is able to modulate adaptive immune responses, of which, especially Th17 responses are implicated in immunopathology. Dectin-1 receptor agonists are also attractive candidates as adjuvants in vaccination against diseases such as tuberculosis and pneumonia where IL-17 provides protective immunity [48]. These ligands may also serve as effective adjuvants in viral diseases and conjugates of antigen-anti-dectin-1 mAb can be used to induce CD4^+^, CD8^+^ T and B cell responses against viral infections.

## Methods

### Stimuli

LPS and Zymosan were obtained from Invivogen (San Diego, CA). They were reconstituted in endotoxin free water at concentrations of 1 mg/ml and 10 mg/ml respectively. Curdlan was obtained from Wako Chemicals (Richmond, VA) and suspended in PBS at 10 mg/ml.

### Isolation and culture of human monocyte-derived DCs

DCs were prepared essentially as described [43]. Peripheral blood mononuclear cells were separated over Ficoll density gradient centrifugation. Cells were allowed to adhere to culture plates for 2 h. Non-adherent cells were removed. The resulting monocytes were cultured under a humidified atmosphere of 5% CO_2_ at 37°C in RPMI 1640 supplemented with 10% FBS, 1 mM glutamine, 100 U/ml penicillin, 100 µg/ml streptomycin, 50 ng/mlhuman rGM-CSF (Peprotech, NJ), and 10 ng/ml human rIL-4 (Peprotech, NJ). Half of the medium was replaced every 2 days with fresh medium and monocyte derived DCs (DC) were collected after 6 days. DCs were cultured with Curdlan (20 µg/ml), Zymosan (40 µg/ml) and LPS (1 µg/ml). After 48 h, the cells were collected for flow cytometry and supernatants were stored for cytokine determination.

### DC phenotype

This was determined by flow cytometry using a FACSCalibur (BD PharMingen, San Diego, CA). Briefly, gated CD14^-^CD11c^+^HLA-DR^+^ DCs were analyzed for the expression of CD40, CD80, CD86, CD83 and HLADR (BD PharMingen) using Flow jo (Treestar Inc).

### Cytokine production by DCs

Cytokines, IL-1β IL-6, IL-12p70 in the supernatants were measured by specific ELISA kits (BD Pharmingen) as per the manufacturer's protocol. IL-23 was measured using an ELISA kit from eBiosciences (San Diego, CA).

### DC-CD4 T cell cultures

Immature DCs were stimulated with LPS, Zymosan and Curdlan as described above. Two days after culture, cells were collected and washed. After washing, 2×10^4^ DCs were cultured with 1×10^5^ purified, naïve allogeneic CD4 T cells (negative selection kit from Stem cell Sep). Seven days later, the supernatant was collected and the secretion of IL-17 (eBiosciences), IL-10 and IFN-γ (BD PharMingen) was assessed using ELISA.

### DC-CD4 T+ B cell cultures for IgG and IgA induction

The culture of DC primed T cells with B cells was essentially as described in the report by Schmitt et al. (44) Briefly, naïve CD4 T cells (1×10^5^) generated as above were cultured with purified, allogenic B cells at a ratio of 1∶1 in 96 well plates. The purity of T cells recovered from DC-T cell culture after 7 days was 85–91% as determined by flow cytometry. Therefore, T cells were used without further purification and B cells were purified by negative selection using magnetic beads (Stem cells, Vancouver). Purity of B cells obtained was above 92%. B cells were activated with IgM (1 µg/ml) and CPG (0.5 µg/ml, ODN2006, Invivogen, San Diego, CA) for 2 h prior to culture with T cells. Two weeks after B-T culture supernatants were collected and assayed for the presence of IgG and IgA in the supernatant by in house ELISA. Nunc maxisorp plates were coated with 1 µg/ml of IgG or IgA (BD Biosciences) overnight at 4°C. After overnight incubation at 4°C, the plates were blocked with PBS containing 10% Fetal bovine serum, washed and incubated with suitable dilutions of the supernatants. Bound Igs were detected using biotinylated detection antibodies (1 µg/ml, BD Biosciences) and HRP-conjugated streptavidin. After washing and addition of substrate, the optical density in the wells was measured at 450 nm and background values were subtracted. The average of duplicate measurements was taken.

For culture of B cells with stimulated DC-Tcell supernatants, purified and activated B cells 1×10^5^(as above) were cultured with supernatants (1∶5-supernatant: media) from LPS, Zymosan and Curdlan stimulated and unstimulated DC-CD4T cultures (these cultures were as described above). After two weeks secretion of IgG and IgA was determined as described.

### DC-CD8T cell cultures for granzyme induction

Immature DCs were stimulated with LPS, Zymosan and Curdlan as described above. Two days after culture, cells were collected and washed. After washing, 2×10^4^ DCs were cultured with 1×10^5^ purified, allogeneic CD8 T cells (negative selection kit from Stem cell Sep). Seven days later, cells were collected and surface-stained for CD8 PerCP. After fixing with 4% paraformaldehyde for 15 min at 37°C, the cells were washed and permeabilized (BD perm buffer). The cells were then stained with antibodies to granzyme B, perforin, Granzyme A and appropriate isotype controls (BD biosciences). Gated CD8 T cells were analyzed for presence of granzyme and perforin.

### DC-CD8T cell cultures for tumor cell lysis

DCs (1×10^6^) were stimulated with LPS, Zymosan, and Curdlan as described above. Two days after culture, cells were collected and washed. After washing, 2×10^4^ DCs were cultured with 1×10^5^ purified, allogeneic CD8 T cell along with 1×10^4^ irradiated PC3 (American Type Culture Collection, Manassas, VA) cells for 10 days to generate PC3 specific target cells. Subsequently, CD8 T cells were purified from the cultures by negative selection using magnetic beads. Purified CD8 T cells (effectors) were incubated with CFSE-labeled PC3 cells (targets) at target: effector ratios varying from 1∶25 to 1∶100 in a volume of 200 µl. Labeling with cell tracking dye CFSE separates the targets from the effectors. Four hours later 5 µl of 7-AAD was added to the cells to stain for dead cells. 7-AAD only enters the membrane of compromised cells and binds to DNA. Cells were acquired on FACS Calibur 10 min after adding 7-AAD. 10,000 CFSE-positive cells were collected per condition. Controls included CFSE-stained PC3 cells without effectors and 7-AAD- and CFSE-stained PC3 cells. Analysis was performed by gating on the target cells and measuring the 7-AAD-negative vs. 7-AAD-positive cells. Cells positive for both 7-AAD and CFSE were considered lysed. Percentage of cytotoxicity was calculated by the following equation: (7-AAD-positive CFSE-positive cells/total number of CFSE-positive cells) ×100.

## Supporting Information

Figure S1 Dose response graph for Zymosan and Curdlan. Concentrations of Zymosan and Curdlan used in the experiments in the manuscript were chosen on the basis of IL-23 secretion. Upper bar graphs represent the levels of IL-12 and IL-23 secreted by DCs in response to various concentrations of Zymosan and Curdlan. Lower graph reresents the level of IL-17 secreted by T cells cultured with various concentrations of Zymosan and Curdlan primed DCs.
(0.08 MB PDF)
