# Supplementary material for: Human Dendritic Cells Activated via Dectin-1 Are Efficient at Priming Th17, Cytotoxic CD8 T and B Cell Responses
Source: PLoS One. 2010 Oct 18;5(10):e13418. doi: 10.1371/journal.pone.0013418 (PMC2956651; doi:10.1371/journal.pone.0013418)
Supplement: Figure S1 — Dose response graph for Zymosan and Curdlan. Concentrations of Zymosan and Curdlan used in the experiments in the manuscript were chosen on the basis of IL-23 secretion. Upper bar graphs represent the levels of IL-12 and IL-23 secreted by DCs in response to various concentrations of Zymosan and Curdlan. Lower graph reresents the level of IL-17 secreted by T cells cultured with various concentrations of Zymosan and Curdlan primed DCs. (0.08 MB PDF) [file pone.0013418.s001.pdf]

Figure S1

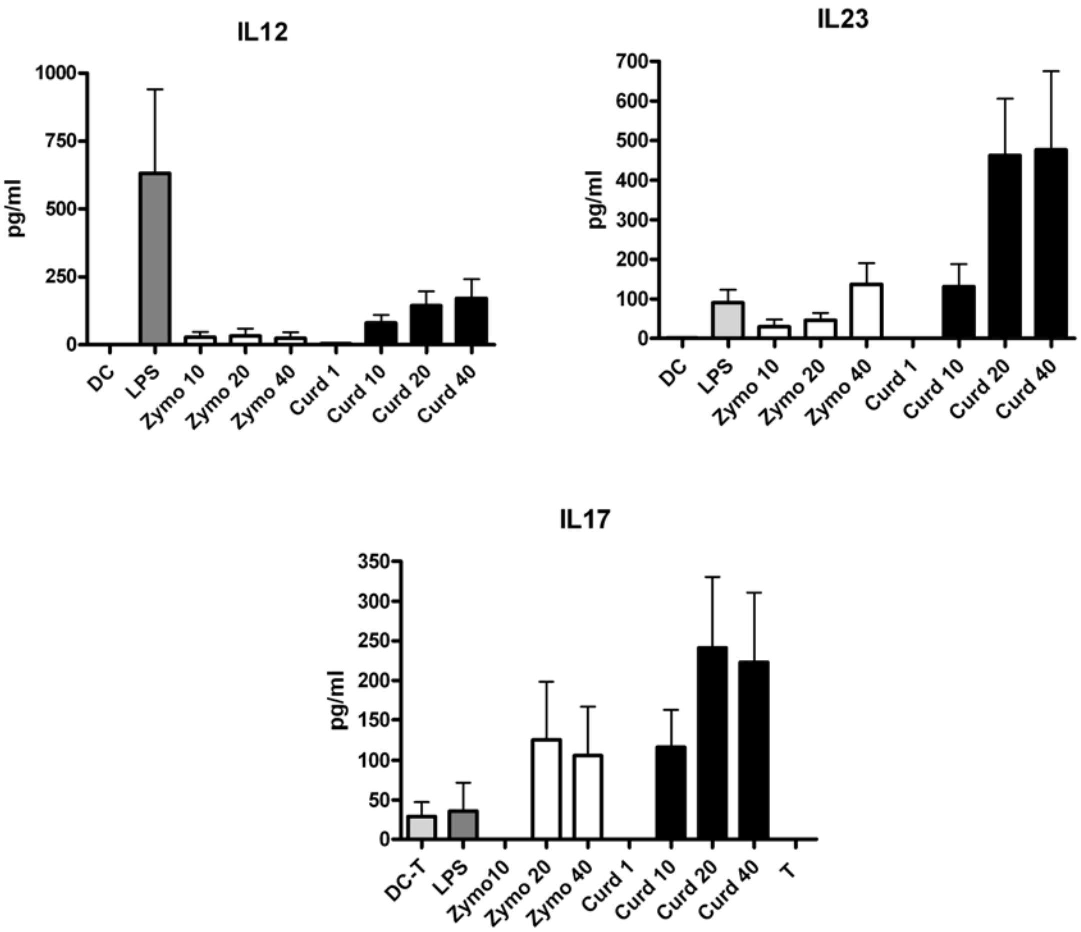

Figure S1: Dose response graph for Zymosan and Curdlan. Concentrations of Zymosan and Curdlan used in the experiments in the manuscript were chosen on the basis of IL -23 secretion. Upper bar graphs represent the levels of IL -12 and IL-23 secreted by DCs in response to various concentrations of Zymosan and Curdlan. Lower graph reresents the level of IL-17 secreted by T cells cultured with various concentrations of Zymosan and Curdlan primed DCs.
